# Clinical Impact of the KL-6 Concentration of Pancreatic Juice for Diagnosing Pancreatic Masses

**DOI:** 10.1155/2015/528304

**Published:** 2015-09-14

**Authors:** Kazuya Matsumoto, Yohei Takeda, Kenichi Harada, Takumi Onoyama, Soichiro Kawata, Yasushi Horie, Teruhisa Sakamoto, Masaru Ueki, Norimasa Miura, Yoshikazu Murawaki

**Affiliations:** ^1^Department of Gastroenterology, Tottori University Hospital, 36-1 Nishi-cho, Yonago 683-8504, Japan; ^2^Department of Pathology, Tottori University Hospital, Yonago 683-8504, Japan; ^3^Department of Surgery, Tottori University Hospital, Yonago 683-8504, Japan; ^4^Center for Promoting Next-Generation Highly Advanced Medicine, Tottori University Hospital, Yonago 683-8504, Japan; ^5^Department of Pharmacotherapeutics, Tottori University Hospital, Yonago 683-8504, Japan

## Abstract

*Background and Aim*. Pancreatic juice cytology (PJC) is considered optimal for differentially diagnosing pancreatic masses, but the accuracy of PJC ranges from 46.7% to 93.0%. The aim of this study was to evaluate the clinical impact of measuring the KL-6 concentration of pancreatic juice for diagnosing pancreatic masses.* Methods*. PJC and the KL-6 concentration measurements of pancreatic juice were performed for 70 consecutive patients with pancreatic masses (39 malignancies and 31 benign).* Results*. The average KL-6 concentration of pancreatic juice was significantly higher for pancreatic ductal adenocarcinomas (PDACs) (167.7 ± 396.1 U/mL) and intraductal papillary mucinous carcinomas (IPMCs) (86.9 ± 21.1 U/mL) than for pancreatic inflammatory lesions (17.5 ± 15.7 U/mL, *P* = 0.034) and intraductal papillary mucinous neoplasms (14.4 ± 2.0 U/mL, *P* = 0.026), respectively. When the cut-off level of the KL-6 concentration of pancreatic juice was 16 U/mL, the sensitivity, specificity, and accuracy of the KL-6 concentration of pancreatic juice alone were 79.5%, 64.5%, and 72.9%, respectively. Adding the KL-6 concentration of pancreatic juice to PJC when making a diagnosis caused the values of sensitivity and accuracy of PJC to increase by 15.3% (*P* = 0.025) and 8.5% (*P* = 0.048), respectively.* Conclusions*. The KL-6 concentration of pancreatic juice may be as useful as PJC for diagnosing PDACs.

## 1. Introduction

Pancreatic cancer is the fifth leading cause of cancer death and has the lowest patient survival rate of any solid cancer. The 5-year survival rate for all patients with pancreatic ductal adenocarcinoma (PDAC) is less than 3.5% [1, 2]. On the other hand, the prognosis of pancreatic inflammatory lesions such as chronic pancreatitis (CP), autoimmune pancreatitis (AIP), and other rare tumors is much better. To differentiate PDAC from these inflammatory conditions is critical, because treatment strategies and prognoses differ. Endoscopic retrograde pancreatography (ERP) is the most commonly used examination for diagnosis and cytology, evaluating the pancreatic juice obtained through a cannula for pancreatography. Pancreatic juice cytology (PJC) is thought to be the most exact diagnostic modality for intraductal papillary mucinous carcinoma (IPMC). However, the accuracy of PJC for PDAC and IPMC has not been satisfactory [3–6], and other modalities are required to improve the accuracy for diagnosing malignancy.

MUC1, membrane-associated mucin, has various types based on different glycoforms in its extracellular domain and is widely expressed in gastrointestinal tissues. Many investigations have shown that aberrant expression of MUC1 in gastrointestinal cancer tissue has clinicopathological and biological importance in cancer disease [7–9]. KL-6 mucin, one kind of MUC1, has also been investigated, and it appears to have a significant relationship with malignant tumor behavior, especially cancer cell invasion and metastasis in various gastrointestinal cancers [7, 8, 10–12].

Inagaki et al. have demonstrated that MUC1 can be used effectively to diagnose intraductal papillary mucinous neoplasms (IPMN) with IPMC; all PDAC specimens were positive on immunohistochemical analysis for KL-6 mucin (unpublished data [13]). Shimamoto et al. reported the usefulness of the quantitative reverse transcription-polymerase chain reaction for MUC1 in pure pancreatic juice for the detection of IPMC [14].

This study extends previous findings by prospectively investigating the clinical benefits of measuring the KL-6 concentration of pancreatic juice from a large number of consecutive patients.

## 2. Patients and Methods

### 2.1. Patients

The Tottori University Hospital Institutional Review Board approved this study involving 70 consecutive patients who underwent PJC for pathological examination of pancreatic masses between October 2011 and December 2012 at the Tottori University Hospital. This study was performed according to the guidelines described in the Helsinki Declaration for biomedical research involving human subjects. All patients provided their written, informed consent for all procedures associated with the study.

The 70 patients with pancreatic disease included 42 men and 28 women, with ages ranging from 36 to 83 years and a mean age of 70.2 years (Table 1). A malignant lesion was present in 39 patients, including 19 men and 20 women, with ages ranging from 45 to 83 years and a mean age of 70.9 years. A benign lesion was present in 31 patients, including 23 men and 8 women, with ages ranging from 36 to 82 years and a mean age of 69.4 years.

### 2.2. Methods

The patients were referred for PJC based on the need to evaluate them for malignancies. Cytodiagnosis of the specimen was performed by Papanicolaou's method.

Pancreatic juice was collected in an inpatient endoscopy suite as previously described [15], using a lateral-viewing endoscope (JF260V; Olympus Optical Co., Ltd, Tokyo, Japan), a cannula (M00535700; Boston Scientific Corporation, Natick, MA, USA), and a 0.035-inch hydrophilic guidewire (M00556051; Boston Scientific Corporation). Over the guidewire, the cannula was advanced into the main pancreatic duct. The guidewire was then withdrawn, and pancreatic juice was collected using a syringe with the tip of the cannula in the MPD. The aspirated material was then evaluated by a cytopathologist (YH).

### 2.3. KL-6 Concentration Measurement

Pancreatic juice was obtained from a pancreatic duct. After pancreatic juice was centrifuged at 1000 rpm for 5 minutes, the cell pellet was subjected to cytological examination. The supernatant (10 *μ*L) was used for measuring the KL-6 concentration. Human KL-6 levels were determined in duplicate with a PICOLUMI KL-6 kit (EIDIA, Tokyo, Japan), an electrochemiluminescence immunoassay (ECLIA) specific for human KL-6.

The immunohistochemical procedures were performed as reported previously [16]. The appropriate dilutions of KL-6 were decided using the pancreatic tissue in the cases of PDAC and IPMC. Pancreatic tissues were obtained by surgery, and, after being treated with 10% buffered formalin, the sliced tissues were embedded in paraffin in the standard manner. Sections, 4 *μ*m thick, were dewaxed and then stained using the following method. Each case was first checked with a hematoxylin and eosin (H&E) stain, and appropriate sections were selected for further immunohistochemical stains. They were incubated with Histofine, Heat Processor Solution pH6 (Nichirei Biosciences Inc., Tokyo, Japan), for 40 min at 100°C and incubated with the monoclonal antibody anti-KL-6 (EIDIA, Tokyo, Japan) at a dilution of 1 : 20,000.

### 2.4. Final Diagnosis

The final diagnosis was determined based on the PJC results, clinical follow-up, and surgical pathology, if available. Patients without a malignant disease, excluding CP, AIP, and IPMN, were followed up by imaging examinations.

All patients were observed closely for immediate or delayed complications. The severity of post-ERCP pancreatitis was determined based on the criteria of Cotton et al. [17].

### 2.5. Data Analysis

Information about all patients undergoing PJC has been prospectively entered into a database since October 2011. The data recorded includes the location, type, size, and endoscopic features of the lesion sampled, sample adequacy, cytology results, final diagnosis, and procedure-related complications.

Diagnostic power between subgroups was compared with the *χ* ^2^ test and the *t*-test. A *P* value less than 0.05 was considered significant. Statistical analysis was performed using IBM SPSS Statistics 21 (IBM JAPAN, Tokyo, Japan).

## 3. Results

Table 1 shows the subjects' characteristics. The malignant group included 34 PDACs and 5 IPMCs, while the benign group included 19 IPMNs and 12 pancreatic inflammatory lesions and benign strictures of the MPD. Both patients with IPMNs and benign pancreatic ductal strictures were followed up by EUS or CT for a mean of 18.7 months (range 13–27 months) but none were found to have a malignant disease.

**Figure 1:**
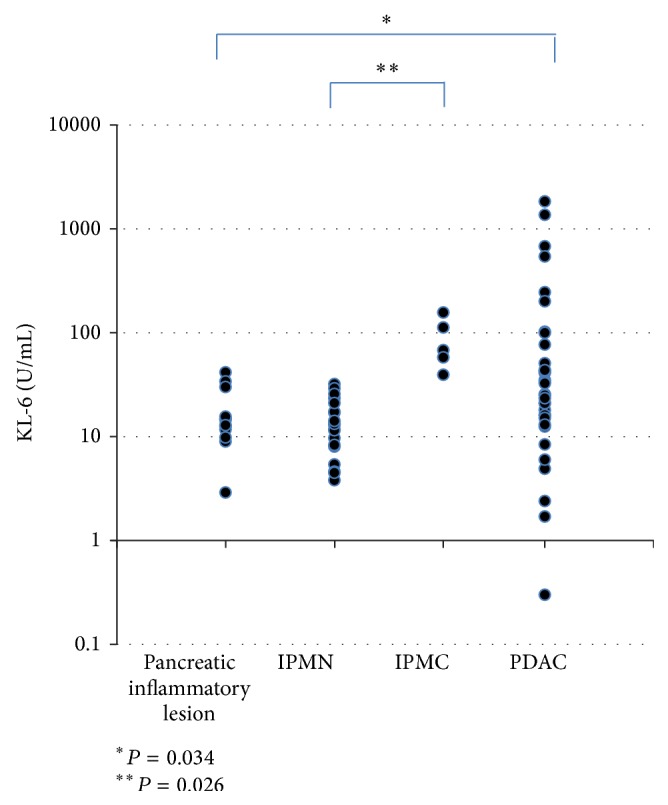
The KL-6 concentrations of pancreatic juice in various pancreatic diseases.

Figure 1 shows the average KL-6 concentration of pancreatic juice in various pancreatic diseases. The average KL-6 concentration of pancreatic juice was significantly higher for PDAC (167.7 ± 396.1 U/mL) than for pancreatic inflammatory lesions and benign strictures of the MPD (17.5 ± 15.7 U/mL, *P* = 0.034). Furthermore, the KL-6 concentration was significantly higher in IPMC (86.9 ± 21.1 U/mL) than in IPMN (14.4 ± 2.0 U/mL, *P* = 0.026).

**Figure 2:**
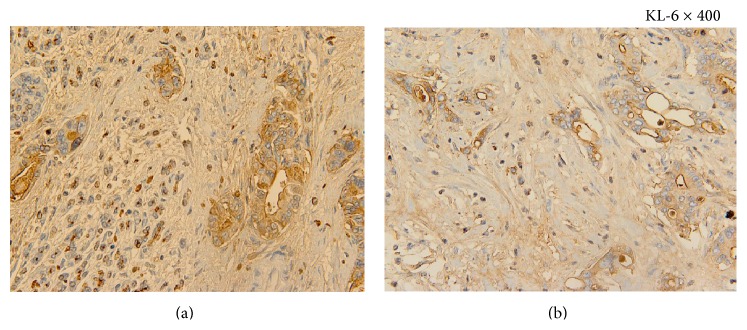
(a) Immunohistochemical staining of KL-6 (KL-6 × 400). KL-6 positivity is observed in the cytoplasm of PDAC cells. (b) Immunohistochemical staining of KL-6 (KL-6 × 400). KL-6 positivity is observed in the cytoplasm of IPMC cells.

Immunohistochemical analysis showed KL-6 positivity in the cytoplasm of PDAC cells (Figure 2(a)) and IPMC cells (Figure 2(b)).

**Figure 3:**
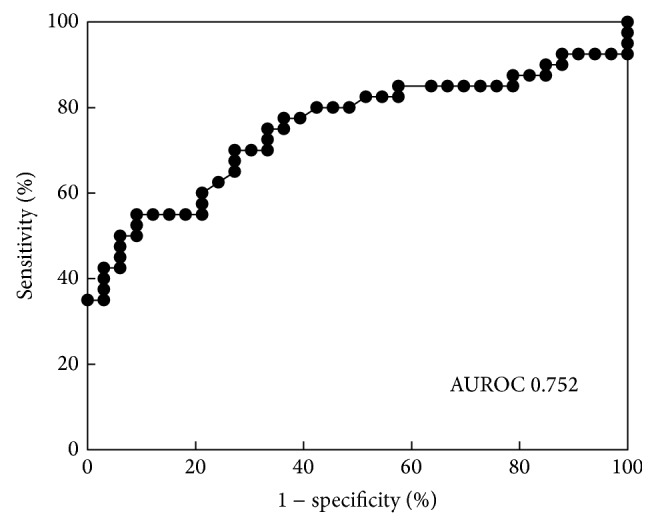
ROC curve of the KL-6 concentration of pancreatic juice for pancreatic malignancy. The cut-off level of KL-6 determined from the receiver-operating characteristic curve for differentiating pancreatic malignancy from benign stricture of the main pancreatic duct and IPMN is 16 U/mL. The AUC of the KL-6 analysis is 0.752.

Figure 3 shows the receiver-operating characteristic (ROC) curve of pancreatic malignancy, which included PDAC and IPMC. The cut-off level of KL-6 was determined to be 16 U/mL for the differentiation of pancreatic malignancy from pancreatic inflammatory lesions and IPMN by the ROC curve. The AUC of the KL-6 analysis was 0.752. When comparing the KL-6 concentration in IPMC with that in IPMN, the ROC curve showed that the optimal cut-off value was from 32.7 to 39.4 U/mL. The AUC of KL-6 analysis was 1.000, an excellent test (data not shown).

Table 2 summarizes the diagnostic ability of PJC and/or KL-6 analysis to differentiate malignant disease (PDAC and IPMC) from benign disease (IPMN and pancreatic inflammatory lesion). The sensitivity, specificity, positive predictive value, negative predictive value, and accuracy of KL-6 concentration of pancreatic juice alone were 79.5%, 64.5%, 73.8%, 71.4%, and 72.9%, respectively, whereas those of pancreatic juice cytology alone were 82.1%, 96.8%, 97.0%, 81.1%, and 88.6%, respectively. Of the remaining 7 patients who remained undiagnosed by cytological assessment, the KL-6 concentration of pancreatic juice was measured in 6 (85.7%). Adding the KL-6 concentration of pancreatic juice to standard cytological assessment increased the sensitivity and accuracy of PJC by 15.3% (*P* = 0.025) and 8.5% (*P* = 0.048), respectively.

Table 3 shows the diagnostic ability of PJC and/or KL-6 analysis for differentiating IPMC from IPMN. The sensitivity, specificity, positive predictive value, negative predictive value, and accuracy of KL-6 concentration alone, and with PJC, were all 100% when the cut-off level of KL-6 concentration was from 32.7 to 39.4 U/mL for differentiating IPMC from IPMN.

Ten patients (14.3%) in this study developed complications following PJC, all of which were mild pancreatitis. All patients were cured with conservative treatment.

## 4. Discussion

Various tumor markers, such as CEA, CA19-9, Span-1, and DUPAN-2, have been widely used for detecting PDAC and IPMC [18]. Several authors have reported that the serum KL-6 concentration was measured in 44% of pancreatic cancer patients [19] and that the KL-6 (MUC1) concentration of PJC specimens can be measured [13, 14, 20]. However, published reports have included relatively small numbers of patients with PDAC and IPMC.

In the present study, a high KL-6 concentration was seen in 79.5% of patients with PDAC and IPMC. The sensitivity and accuracy of pancreatic juice cytology for the diagnosis of PDAC and IPMC were significantly improved by adding the KL-6 concentration. Among 7 cases whose PJC results were inconclusive or negative, 6 had elevated KL-6 concentrations in pancreatic juice and were finally diagnosed as having PDAC and IPMC. These findings indicate that the KL-6 concentration of pancreatic juice is clinically useful when PJC specimens are inadequate, making differentiation between malignant and benign conditions difficult. In addition, the KL-6 concentration is useful for discriminating IPMC from IPMN, because the AUC of the KL-6 concentration of pancreatic juice was 1.000. Thus, when IPMN is suspected based on the clinical course or imaging findings, the KL-6 concentration of pancreatic juice may be helpful to exclude IPMN.

The measurement of KL-6 concentration did not affect the diagnostic power of PJC because the KL-6 concentration was checked using the pancreatic juice from which the cell pellet was removed for cytological examination.

Previous reports have shown that PJC has yielded sensitivities for pancreatic cancer that ranged from 33.3% to 67%, with specificity of 100%, PPV of 100%, NPV of 27.3% to 98%, and accuracy of 46.7% to 94% [4, 15, 21, 22]. A recent study showed the usefulness of PJC for pancreatic-ductal strictures, with sensitivity of 71.4% to 93%, specificity of 100%, PPV of 100%, NPV of 75% to 84.4%, and an accuracy of 88.8% to 94% [15, 22]. In the present prospective study, PJC showed excellent diagnostic ability for malignant pancreatic tumors [15].

The major complication of procedures associated with PJC is pancreatitis. In the present series, 10 patients (14.3%) developed mild pancreatitis after PJC; thus, we must restrict PJC to when we cannot acquire the evidence by endoscopic ultrasound-guided fine needle aspiration biopsy.

In conclusion, the KL-6 concentration of pancreatic juice strengthened the diagnostic ability of PJC for pancreatic tumors.

## Figures and Tables

**Table 1:** Patients' characteristics.

	Pancreatic inflammatory lesion	IPMN	IPMC	PDAC
Number of patients (M/F)	12 (8/4)	19 (15/4)	5 (1/4)	34 (18/16)
Mean age, y (range)	65.6 (36–80)	71.7 (55–82)	79.6 (77–83)	69.9 (45–83)
Mean size of mass, mm (range)	—	30.0 (4–60)	28.2 (20–50)	31.6 (6–56)
Tumor marker (serum, SD)				
CEA	2.8 ± 2.3	3.6 ± 2.8	2.1 ± 2.0	21.4 ± 70.5
CA19-9	10.4 ± 9.0	9.3 ± 30.9	17.9 ± 17.3	1534.0 ± 3729.2^a^
Span-1	7.1 ± 5.9	12.5 ± 10.1	38.8 ± 41.6	935.0 ± 4163.4
DUPAN2	33.4 ± 30.8	59.1 ± 80.6	53.6 ± 55.0	2404.7 ± 10451.1
KL-6	249.2 ± 121.9	294.6 ± 210.3	280.4 ± 262.7	453.6 ± 503.7^b^

^*^Materials of which final diagnosis was obtained by operation or clinical follow-up.

^a^ *P* = 0.017 compared with pancreatic inflammatory lesion.

^b^ *P* = 0.027 compared with pancreatic inflammatory lesion.

**Table 2:** Diagnostic ability of PJC and/or KL-6 measurement of pancreatic juice for differentiating pancreatic malignancy from pancreatic inflammatory lesion and IPMN.

PDAC or IPMC (*n* = 39) and IPMN or pancreatic inflammatory lesion (*n* = 31)					
	Sensitivity, %	Specificity, %	PPV, %	NPV, %	Accuracy, %
KL-6 measurement	79.5	64.5	73.8	71.4	72.9
	(31/39)	(20/31)	(31/42)	(20/28)	(51/70)

PJC	82.1	96.8	97.0	81.1	88.6
	(32/39)	(30/31)	(32/33)	(30/37)	(62/70)

PJC and KL-6 measurement combined	97.4^a^	96.8	97.4	96.8	97.1^b^
	(38/39)	(30/31)	(38/39)	(30/31)	(68/70)

^*^Materials of which final diagnosis was obtained by operation or clinical follow-up.

^a^ *P* = 0.025 compared with cytopathology alone.

^b^ *P* = 0.048 compared with cytopathology alone.

**Table 3:** Diagnostic ability of PJC and/or KL-6 measurement of pancreatic juice for differentiating IPMC from IPMN.

IPMC (*n* = 5) and IPMN (*n* = 19)					
	Sensitivity, %	Specificity, %	PPV, %	NPV, %	Accuracy, %
KL-6 measurement	100	100	100	100	100
	(5/5)	(19/19)	(5/5)	(19/19)	(24/24)

PJC	100	100	100	100	100
	(5/5)	(19/19)	(5/5)	(19/19)	(24/24)

PJC and KL-6 measurement combined	100	100	100	100	100
	(5/5)	(19/19)	(5/5)	(19/19)	(24/24)

^*^Materials of which final diagnosis was obtained by operation or clinical follow-up.
